# Delivering sustainable food: exploring college students’ willingness to pay for carbon labels in China via a choice experiment

**DOI:** 10.3389/fpubh.2026.1910782

**Published:** 2026-09-17

**Authors:** Jie Zhou, Zhiyi Cai, Dungang Zang, Jian Zhang

**Affiliations:** 1 College of Economics, Sichuan Agricultural University, Chengdu, Sichuan, China; 2 College of Economics, Northwest Normal University, Lanzhou, Gansu, China

**Keywords:** carbon labels, discrete choice experiment, environmental information disclosure, online food delivery platforms, sustainable food delivery, willingness to pay

## Abstract

The rapid expansion of online food delivery has intensified concerns about packaging waste and carbon emissions, making sustainable consumption an increasingly important component of the sector’s low-carbon transition. Whether environmental information disclosure can shift consumers’ choices toward lower-carbon alternatives is therefore an important issue for improving the sustainability of digital food-delivery platforms. This study estimates the preferences of university students, who constitute one of the most intensive user groups of online food-delivery services, and willingness to pay (WTP) for carbon-footprint information and packaging materials on online food-delivery platforms. Data from 692 surveys conducted among university students in the Sichuan-Chongqing region in August 2023 were analyzed using a discrete choice model. This analysis examined how the disclosure of carbon footprint (CF) and other environmental attributes affects consumer preferences. Key findings include: Firstly, compared with high-carbon-footprint takeaways, university students show a significant positive preference for low-carbon-footprint alternatives, with an estimated WTP of an additional $1.65 per order. Meanwhile, consumers also prefer takeaways with environmentally friendly packaging. Secondly, male students, those with a proactive environmental stance, and those with greater awareness of environmental knowledge were more inclined to opt for takeaways with lower carbon footprints. Greater environmental knowledge also led to a preference for eco-friendly packaging options. The findings underscore the critical role of carbon label disclosures in steering university students towards environmentally friendly choices in food delivery, thereby charting a feasible pathway towards achieving low-carbon goals. This aligns with the promotion of efficient takeaway dietary habits among young generations.

## 1 Introduction

The take-out food sector, an emerging online-to-offline (O2O) industry that facilitates the delivery of meals from physical restaurants to consumers ordering online, significantly influences the daily lives of most urban residents and has attracted growing scholarly attention (1, 2). As mobile Internet penetration in China expands and the pace of consumers’ lives accelerates, demand for dining convenience is rising. Notably, during the COVID-19 pandemic, take- out demand surged, driving a rise in online orders (3, 4). In the post-COVID-19 era, offering delivery services has become standard practice for restaurants (5). Regarding the size of online food delivery users, the latest available data show that as of December 2023, this figure in China had reached 545 million, comprising nearly half (49.9%) of the country’s internet users (6). In terms of market penetration, online food delivery’s share of China’s total restaurant spending increased from 11 to 25% between 2018 and 2022 (7), reflecting a steady increase in the service’s consumer penetration. While take-out services provide convenience, they also entail substantial use of disposable utensils, higher energy consumption, and packaging waste, contributing significantly to carbon emissions. In terms of environmental impacts, the issue of carbon emissions from take- out is particularly acute in China, where the top three restaurant platforms process 20 million orders daily, using around 60 million plastic items (3). Each take-out order produces approximately 111.80 grams of carbon dioxide equivalent, with most emissions (86%) deriving from food packaging (8). As the world’s leading emitter of carbon dioxide, China is under significant pressure to reduce its greenhouse gas emissions (9–11). However, given China’s resource characteristics of being “coal-rich, oil-scarce, and gas-poor,” adjusting the energy structure is a gradual process, and the rising energy demand driven by industrialization and urbanization might conflict with economic growth (12). Consequently, modifying consumer behavior is crucial for achieving energy efficiency and reducing emissions in China (13–15).

As key future consumers and opinion leaders, college students’ choices and environmental consciousness will significantly influence society. Recently, online take-out ordering has gained substantial popularity among China’s youth (16), becoming a regular part of their daily lives (17). Take-out services have evolved beyond simple meal delivery, reflecting significant shifts in modern lifestyles and consumption patterns. With the widespread adoption of internet technology and digital environments, particularly through mobile payments, consumers, exemplified by Meituan, which recorded a daily peak of 78 million orders in the third quarter of 2023, are increasingly adopting take-out habits. Managing carbon emissions while leveraging the convenience of take-out services is now a societal priority. Research by He et al. (18) on the Eleme platform showed that setting “no utensils” as the default option and rewarding green points increased the proportion of utensil-free orders by 648%. This study underlines the potential of carbon label prompts to encourage college students to choose environmentally friendly options like low-carbon take-out and biodegradable utensils, thus maintaining the convenience and appeal of take-out culture. Understanding these consumption behaviors could lead to effective strategies for promoting sustainable consumption and reducing societal carbon footprints. This insight not only indicates future consumption trends but also aids businesses in strategy development, fosters sustainable consumption, supports the crafting of environmental policies, and facilitates green societal progress.

Recent years have seen a growing focus on food carbon-labeling research, predominantly in developed nations such as the USA and Germany, with fewer studies addressing developing countries (19–21). In China, carbon labeling remains in its nascent stages, mainly applied to electronics and services, but has not yet been adopted in the take-out sector. Research on consumers’ willingness to buy carbon-labeled take-out food has significant environmental implications. Indeed, given the substantial environmental burden imposed by the take-out industry, the Chinese government launched the “Green Take-out Management Standards” in 2023. These standards aim to reduce carbon emissions from take-out services by promoting low-carbon principles, reducing packaging use, and encouraging eco-friendly materials. This initiative requires the take-out sector to re-evaluate and refine its operational practices to decrease emissions. For the take-out industry to evolve sustainably and eco-consciously, it is crucial to encourage consumers to make low-carbon choices. Carbon labels play a vital role here, attracting global attention as a key mechanism for fostering green consumption (22). These labels detail the greenhouse gas emissions throughout a product’s life cycle, guiding consumers toward products that are both low in carbon and environmentally benign, thereby facilitating more sustainable consumption practices (23). This approach not only engages consumers in energy saving and emission reduction but also strengthens their capacity to make environmentally sound choices. Furthermore, consumer preferences can drive manufacturers to produce less polluting products, thereby reinforcing their commitment to environmental stewardship. Thus, understanding consumer readiness to opt for carbon-labeled products is crucial for enhancing the sustainability of our environmental resources.

The take-out market in China is vast, and studying consumer preferences for carbon-labeled take-out food and their willingness to pay (WTP) not only has practical significance but can also have a global impact. Currently, China is the largest take-out market globally, accounting for 60% of global market revenue, excluding phone orders (24). Compared to other countries, China’s take-out market reached 1.1 trillion yuan in 2022 (25), far exceeding the UK (£5746.4 million) and Italy (£7240.5 million), more than 10 times their size (26). The enormous market size and large consumer base make China the most appropriate place to conduct this research. At the same time, this paper will use the choice experiment method to assess the willingness to pay (WTP) of college student consumers for carbon-labeled take-out, which has two major methodological advantages: Firstly, the choice experiment method is rarely used in related studies in China, which cannot compare consumer preferences for low-carbon attributes and other attributes, as well as the interaction between low-carbon attributes and other attributes, and using the choice experiment method will overcome this shortcoming and fill the related literature gap; Secondly, since carbon labels have not yet been implemented in take-out services, the choice experiment method is particularly effective for evaluating hypothetical scenarios of attributes or product feature combinations not yet present in the market.

In this study, a discrete choice experiment was conducted using 692 valid questionnaires collected from college students in the Sichuan-Chongqing region of China. The experiment incorporated five attributes: price, platform rating, delivery time, carbon footprint, and packaging material. The first three capture conventional economic and service considerations in online food-delivery choices, whereas carbon footprint and packaging material capture the environmental dimensions central to this study. Meanwhile, using conditional logit, mixed logit, and latent class models, the study analyzes Chinese college students’ preferences and willingness to pay for carbon-related attributes of online food delivery and further assesses the potential of carbon-footprint labelling to encourage lower-carbon choices. Specifically, the conditional and mixed logit models are used to estimate attribute preferences and willingness to pay, with the mixed logit model additionally capturing continuous preference heterogeneity, while the latent class model is employed to identify distinct consumer segments with different trade-offs among environmental, economic, and service attributes. At the same time, it also examines whether consumers are willing to pay a premium for environmentally friendly take-out packaging, as well as their preferences for other traditional attributes. Additionally, the sources of heterogeneity in consumer preferences for take-out carbon labels are explored in terms of individual characteristics, environmental attitudes, and subjective environmental knowledge. Based on the latent class model, consumers are categorized into four types with distinct preference characteristics: high sensitivity to carbon attributes, price-quality priority, cost-effective and environmentally oriented, and low-carbon consumption types, thereby contributing to and extending the existing literature. The specific contributions of this paper are reflected in the following aspects.

First, most existing studies focus on nutrition- and safety-related food labels, such as nutritional and organic labels. Few studies measure and label the carbon footprint (CF) of food production, and carbon labeling has not yet been implemented in Chinese take-out services. This paper deeply analyzes the factors influencing consumer preferences for carbon-related attributes of carbon-labeled take-out and their heterogeneity, using a latent class model to reveal significant differences in choice preferences between different consumer groups. This is crucial for understanding consumer needs and preferences and provides a more comprehensive set of promotional suggestions or more accurate guidance for governments, take-out platforms, and take-out vendors.

Second, research on food carbon labels is mainly concentrated in developed countries, while studies on developing countries are less frequent (12). This paper, by assessing the willingness to pay (WTP) of Chinese university students for different attributes of carbon-labeled take-out food, significantly deepens the understanding of consumer preferences for carbon labels in developing countries, broadens the literature about carbon footprint, and provides a specific case study for subsequent research, expanding the geographic and cultural boundaries of carbon label research.

Furthermore, Liu et al. (27) found that consumer preferences for low-carbon products can benefit supply chain enterprises. This paper, by exploring consumer preferences for different levels of carbon labels and environmentally friendly packaging materials in take-out food, as well as their willingness to pay, provides a scientific basis for pricing low-carbon products in the take-out industry and for business decision-making. It not only enriches the theoretical framework but also provides practical guidance for the low-carbon transformation of take-out enterprises.

Finally, the empirical results of this study provide empirical evidence for the low-carbon transformation of the take-out industry and for the formulation and implementation of carbon label policies. It proposes a multi-agent, diversified plan to support the implementation of carbon label policies at multiple levels-government, take-out platforms, merchants, and consumers-and provide suggestions for effective collaboration between various stakeholders to enhance the operability of carbon label policies. This also provides strategic support for fostering consumers’ low-carbon consumption habits and the sustainable development of the take-out industry. The research contributes to raising public awareness of low-carbon consumption and encouraging lower-carbon choices in online food delivery, thereby supporting broader efforts to reduce consumption-related carbon emissions and promote sustainable consumption.

The primary objective of this study is to assess whether the disclosure of carbon-related information influence college students’ online food-delivery choices and to quantify the monetary value they attach to lower-carbon attributes. Specifically, this research addresses four research questions. RQ1: Do college students prefer food-delivery options displaying lower carbon-footprint levels and environmentally friendly packaging, and how much are they willing to pay for these attributes? RQ2: How do students’ valuations of carbon-related attributes compare with their valuations of conventional service attributes, particularly platform ratings and delivery time, after accounting for price? RQ3: To what extent do sociodemographic characteristics, environmental attitudes, and subjective environmental knowledge explain heterogeneity in these preferences? RQ4: Do distinct preference segments exist among college students, and, if so, what are their relative sizes and sociodemographic and attitudinal profiles? By addressing these questions, the study evaluates the potential of carbon-footprint disclosure as a demand-side instrument for promoting more sustainable food-delivery consumption.

The remaining sections of this article are structured as follows: Section 2 is a literature review. Section 3 explains the design of the experiment, data collection processes, and the methodology. Section 4 presents the empirical results. Section 5 shows the research discussion. The final section summarizes the main findings and promotes policy implications.

## 2 Literature review

Numerous studies have rigorously quantified the carbon footprint associated with takeaway services. According to Xie et al. (8), significant emissions arise at various phases, including the production of raw materials, meal preparation, packaging, and the transportation process, with the carbon dioxide emissions equivalent(CO_2_-eq) of an average takeaway order reaching 111.80 grams. Camps-Posino et al. (28) estimate that, in the absence of mitigative actions, takeaway packaging in China is responsible for approximately 13 million tons of CO2 equivalents. The “Research Report on the Public’s Low-Carbon Lifestyle under Internet Platforms” suggests that eschewing disposable chopsticks could cut emissions by 16 g CO₂ per use, and adopting reusable utensils can slash waste production by up to 92%, alongside significant reductions in environmental emissions and water use (29). On the transportation front, Norberg-Hodge et al. (30) identify the transportation of food over long distances as a primary contributor to fuel-related pollution and greenhouse gas emissions. USEPA data reveals that an average delivery vehicle for takeaway services emits 4.6 tons of greenhouse gases annually. Xie et al. (8) further demonstrate that opting to walk to nearby restaurants (<1 km) for meal collection can diminish CO₂ emissions associated with takeaway delivery by 68%, underscoring the critical role of consumer behavior modification in mitigating environmental impacts.

While discussing the carbon footprint associated with takeaway services, examining consumers’ preferences for low-carbon options and the factors influencing these preferences is equally vital. Fishbein and Ajzen (31) have indicated that an individual’s consumption behavior is influenced by their attitude towards that behavior, which in turn is related to their behavioral intentions. Environmental attitudes are considered significant in affecting an individual’s eco-friendly actions (32–34). Laroche et al. (35) discovered that an individual’s environmental attitude not only impacts their choices of green products but also enhances their willingness to pay more for eco-friendly products. The confidence in the positive environmental impact of purchasing green products significantly encourages their intentions to make such purchases (36–38). Furthermore, environmental knowledge is often seen as another crucial factor influencing pro-environmental behavior. The prevailing assumption is that if a person lacks awareness of environmental issues, they are less likely to be consciously concerned about the environment and engage in thoughtful pro-environmental actions (39, 40). Hence, according to Frick et al. (41), environmental knowledge is typically viewed as a prerequisite for autonomous action. However, some studies argue that there is no direct link between environmental knowledge and pro-environmental behavior (42). Researchers have also extensively differentiated between subjective (or self-reported) knowledge (i.e., perceived knowledge), actual (or objective) knowledge (i.e., what people truly know), and personal experience (43). Most studies find that objective environmental knowledge can only influence pro-environmental behavior through the complete mediating role of environmental attitudes (44), or can only directly predict a few types of pro-environmental behaviors (45), while subjective environmental knowledge can not only predict pro-environmental behavior through the mediating role of environmental attitudes but also has a direct predictive effect (44, 46–48). Empirical studies further suggest that subjective environmental knowledge may influence pro-environmental behavior more strongly than objective knowledge (43, 49). Taken together, these findings indicate that environmental attitudes and subjective environmental knowledge are important sources of heterogeneity in pro-environmental choice.

Beyond these individual-level determinants, a growing body of research has examined consumers’ direct responses to carbon information and carbon labels. Recent studies have increasingly used discrete choice experiments (DCEs) to investigate consumer preferences and willingness to pay for carbon-related and other sustainability attributes. These studies generally show that consumers value environmental information, although the magnitude of such preferences varies across products, information conditions, and market contexts (21, 50, 51). Evidence from food-choice settings points in a similar direction. Lohmann et al. (52) and Beyer et al. (53) showed that carbon-footprint information can shift choices towards lower-emission alternatives, whereas Casati et al. (54) found that the effectiveness of carbon labels depends on how consumers are exposed to and process the information. Hartikainen et al. (55) further showed that consumers may have difficulty interpreting carbon-footprint information and tend to value labels that facilitate comparisons across products. Importantly, these findings also suggest that consumer responses to sustainability information are unlikely to be homogeneous. To further capture this heterogeneity, previous choice-experiment studies have employed latent class models to identify groups with distinct preferences for environmental labels and to examine how consumer characteristics are associated with class membership. For example, Peschel et al. (56) identified heterogeneous consumer segments in responses to carbon- and water-footprint labels across Canada and Germany, while Broeckhoven et al. (57) used latent class modelling to distinguish consumer groups with different valuations of carbon-labelled food products. Taken together, these studies demonstrate the value of DCEs for quantifying attribute-specific preferences and willingness to pay, while also showing that latent class modelling can reveal distinct preference patterns that may be obscured by average estimates.

Evidence from China remains more limited and is concentrated outside the online food-delivery sector. Zhao et al. (58), using a consumer survey in Chengdu, documented relatively low awareness and understanding of carbon labels, despite generally positive purchase intentions. Xu and Lin (12) similarly found that consumers’ understanding of and trust in carbon labels were positively associated with their willingness to pay a premium for carbon-labelled products. More recent evidence from agricultural products also indicates substantial willingness to pay for carbon-labelled alternatives (59). These findings suggest that Chinese consumers may value carbon information positively, but limited familiarity with carbon labels and sensitivity to price premiums can constrain the extent to which such preferences translate into actual choices. They also provide a direct rationale for giving respondents a brief explanation of carbon-footprint information before the choice tasks in the present study.

Online food-delivery choices are inherently multi-attribute, involving simultaneous trade-offs among monetary cost, service quality, convenience, and other product-related characteristics. Accordingly, previous research has increasingly incorporated attributes such as price, delivery time, restaurant ratings, and packaging quality when examining consumers’ preferences and willingness to pay for online food-delivery services (60). Specifically, price captures the monetary cost of an order and provides the basis for estimating willingness to pay, while delivery time reflects the convenience and time costs associated with food delivery. Platform ratings provide a readily observable signal of restaurant and service quality, and real-world evidence shows that such ratings systematically influence restaurant and online food-delivery choices (61). Packaging material was included as an environmental attribute because packaging constitutes a major source of food-delivery emissions; Xie et al. (8) estimate that it accounts for approximately 86% of the carbon footprint of an average delivery order. Considering these attributes jointly therefore allows carbon-related preferences to be evaluated against the conventional trade-offs consumers face when ordering food online.

In summary, the existing literature generates several expectations for the present study. First, lower-carbon-footprint options and environmentally friendly packaging are expected to generate positive consumer utility and willingness to pay relative to less sustainable alternatives. Second, although carbon-related attributes are expected to influence food-delivery choices, their importance is likely to coexist with conventional considerations such as price and service quality. Third, previous evidence on environmental knowledge and attitudes suggests that consumers with greater subjective environmental knowledge and more pro-environmental attitudes are likely to place greater value on carbon-related attributes. Finally, the substantial variation documented across consumers and market contexts suggests that these preferences are unlikely to be homogeneous, providing a basis for examining distinct preference segments.

This research innovates in several key areas: Firstly, it focuses on the specific group of college students, supplementing previous studies since college students, as a major consumer force of takeaway services, have a significant impact on market trends. Secondly, despite the rich research achievements of foreign scholars in the method of discrete choice experiments, there remains a research gap in the context of China, especially regarding the application in the takeaway industry. This paper fills this gap with empirical research in the Sichuan-Chongqing area of China, providing a comparative analysis of consumer preferences for carbon-related attributes and other attributes of takeaway food, as well as an in-depth discussion on the sources of consumer preference heterogeneity. Lastly, while existing studies have found that information can affect consumer behavior (62, 63), few have considered the impact of external information stimuli on consumer behavior regarding low-carbon consumption. Given that carbon labeling has not been widely implemented in China’s takeaway industry, this study effectively tests the impact of carbon footprint information disclosed by carbon labels on consumer choices using the discrete choice experiment method.

## 3 Research design and methodology

### 3.1 The study sample

During August 2023, online questionnaires were distributed to college students in the Sichuan-Chongqing region through social media and school forums. Respondents were drawn from a range of institutions, with the largest shares coming from Sichuan University (22.4%), Sichuan Agricultural University (15.8%), Southwest University (14.2%), and Sichuan Normal University (9.2%); the remainder were distributed across other universities in the region (see Supplementary Figure S1). To broaden coverage and improve the response rate, volunteers at participating universities also reposted the questionnaire on campus online platforms.

The Sichuan-Chongqing region was selected because it offers a relevant inland setting for examining college students’ online food-delivery choices. Chengdu and Chongqing are major food-consumption and online food-delivery markets in western China, and the region also has a substantial higher-education base. The surveyed institutions spanned different university types and academic disciplines, providing a heterogeneous student sample for examining variation in preferences for carbon-related food-delivery attributes. As an inland metropolitan setting, the region also complements the existing carbon-labelling literature, which has largely focused on developed countries and more developed urban contexts.

To ensure survey quality, the questionnaire was pretested with 30 college students who had experience using online food-delivery services. Participants first completed the full questionnaire, including the choice tasks, and were then invited to provide feedback on the clarity of the instructions and wording, the presentation of the carbon-footprint information, and the comprehensibility and difficulty of the choice tasks. Based on this feedback, ambiguous wording and the presentation of the choice tasks were refined before formal data collection. The 30 pretest responses were not included in the final analytical sample. Several measures were used to ensure data quality during the formal survey. Screening questions on weekly food-delivery frequency and the proportion of staple food in food-delivery consumption were used to retain respondents for whom the choice tasks were behaviorally relevant. The Questionnaire Star platform employed IP-based checks to reduce duplicate submissions. After data collection, questionnaires completed in substantially less time than average and responses showing uniform answering patterns across different questions were excluded. After these screening procedures, 692 valid questionnaires remained for analysis.

### 3.2 Experimental design

Before presenting the choice experiment in questionnaire form, it’s necessary to first determine the number of product attributes and levels, and construct the set of choice tasks.

In setting the product attributes and levels, this study considered that university student consumers would theoretically take into account all the attributes of takeout food and their levels, and then make a decision between two alternative options and not choosing any. Therefore, the setting of attributes and their levels should be kept moderate to avoid too many and lead to consumers making choices based solely on price. The process was mainly based on relevant literature and further refined with reference to previous DCE applications and input from researchers and relevant stakeholders (64, 65). Five attributes were retained to limit the cognitive burden of the choice tasks, consistent with the relatively small attribute sets commonly used in applied DCEs (66). In addition, the study also referred to carbon footprint labeling and the use of eco-friendly packaging as carbon reduction measures, especially incorporating carbon labeling into the study, as it has not yet been applied in the Chinese takeout industry, representing an academically valuable research direction. Since the carbon label serves as a tool to display the carbon footprint of takeout food in the form of a label, this paper includes the takeout carbon footprint as one of the study attributes. Eventually, this study identified five attributes: payment price, platform rating, delivery time (traditional attributes), and carbon footprint and meal box packaging material (carbon-related attributes), with the classification aimed at comprehensively assessing the importance consumers place on both the consumption value and environmental value of takeout food.

The levels of the five attributes were specified as follows. For the traditional service attributes, four price levels (¥13, ¥17, ¥21, and ¥25) were selected based on prevailing prices on online food-delivery platforms during the survey period. These prices were converted to USD based on the exchange rate at the commencement of the survey, equating to $1.82, $2.38, $2.94, and $3.50, respectively. Delivery time was classified as shorter (≤30 min) or longer (>30 min). Platform rating was divided into poor (<4.3), fair (4.3–4.7), and excellent (≥4.7), reflecting the relatively compressed rating distribution commonly observed on Chinese food-delivery platforms.

For the environmental attributes, carbon footprint was presented using a traffic-light format—low (green), average (yellow), and high (red)—to facilitate interpretation (23). Drawing on Xie et al. (8), who estimated food-delivery carbon footprints for different Chinese city tiers, the average level was set at approximately 100 gCO_2_-eq per order, with low and high levels set at 60 and 160 gCO_2_-eq, respectively. These values were intended to provide plausible and clearly differentiated emission levels for the choice tasks. Packaging material included three alternatives: disposable, recyclable, and degradable, allowing consumers’ valuations of different packaging options to be distinguished. Table 1 summarises all attributes and their corresponding levels.

**Table 1:** Attributes and levels of discrete choice experiments.

Attribute category	Attributes	Levels
Traditional attributes	Price	A. ¥13 ($1.82); B. ¥17 ($2.38); C. ¥21 ($2.94); D. ¥25 ($3.5)
	Platform ratings	A. Poor (<4.3); B. Fair (4.3 ~ 4.7); C. Excellent (≥4.7)
	Delivery time	A. Shorter (≤30 min); B. Longer (>30 min)
Carbon related attributes	Carbon footprint	A. Low carbon emissions; B. Average carbon emissions; C. High carbon emissions
	Packaging materials	A. Disposable; B. Recyclable; C. Degradable

In the process of finalizing the choice sets, based on the configurations outlined in Table 1, the implementation of a full factorial design would result in 216 hypothetical scenarios. Given the extensive number of attributes and levels involved, the study employs a fractional factorial design to streamline the selection, consequently reducing the assortment of choice sets to a more manageable 12. To reduce the burden on participants and uphold the integrity of the survey outcomes, the questionnaires were divided into two versions, A and B, each comprising six distinct choice sets. This bifurcation was specifically aimed at the university student demographic, ensuring participants engaged with either version A or B, necessitating six selections per respondent. This stratagem was meticulously designed to prevent any overlap between the respondent groups of the two questionnaire variants, thereby guaranteeing the purity of data collection. Within each choice scenario, participants were tasked with deciding between two offered takeout service options or selecting an exit alternative, a methodological choice designed to authentically capture their preferences.

The questionnaire is divided into four parts: (1) Demographic variables (age, gender, school, and major, monthly living expenses); (2) Screening questions to filter valid subjects, filtering out eligible respondents through “frequency of takeout consumption” and “proportion of staple food in takeout consumption”, eligible respondents continue to complete the third and fourth parts of the questionnaire, otherwise, the questionnaire ends; (3) Choice task set (as shown in Figure 1), setting consumption scenarios, making subjects choose takeout food, each subject needs to make 6 choices; (4) Drawing on existing research, this questionnaire adopts the New Ecological Paradigm (NEP) scale by Dunlap et al. (67) and the Subjective Environmental Knowledge Scale by Tang et al. (68) (as shown in Table A1), using 5-point and 7-point Likert scales respectively, with endpoint labels “strongly agree” and “strongly disagree”, to assess participants’ scores in “environmental attitudes” and “subjective environmental knowledge”. Specifically, the New Ecological Paradigm scale is the most widely used environmental attitude scale globally (69), covering five important aspects: views on natural balance (items 3, 8, 13), anthropocentrism (items 2, 7, 12), human exceptionalism (items 4, 9, 14), ecological crisis (items 5, 10, 15), and limits to growth (items 1, 6, 11), with odd-numbered items scored positively and even-numbered items scored inversely; while the Subjective Environmental Knowledge Scale of Tang et al. (68) includes dimensions of subjective everyday environmental knowledge (items 11–15) and subjective professional environmental knowledge (items 1–10).

**Figure 1:**
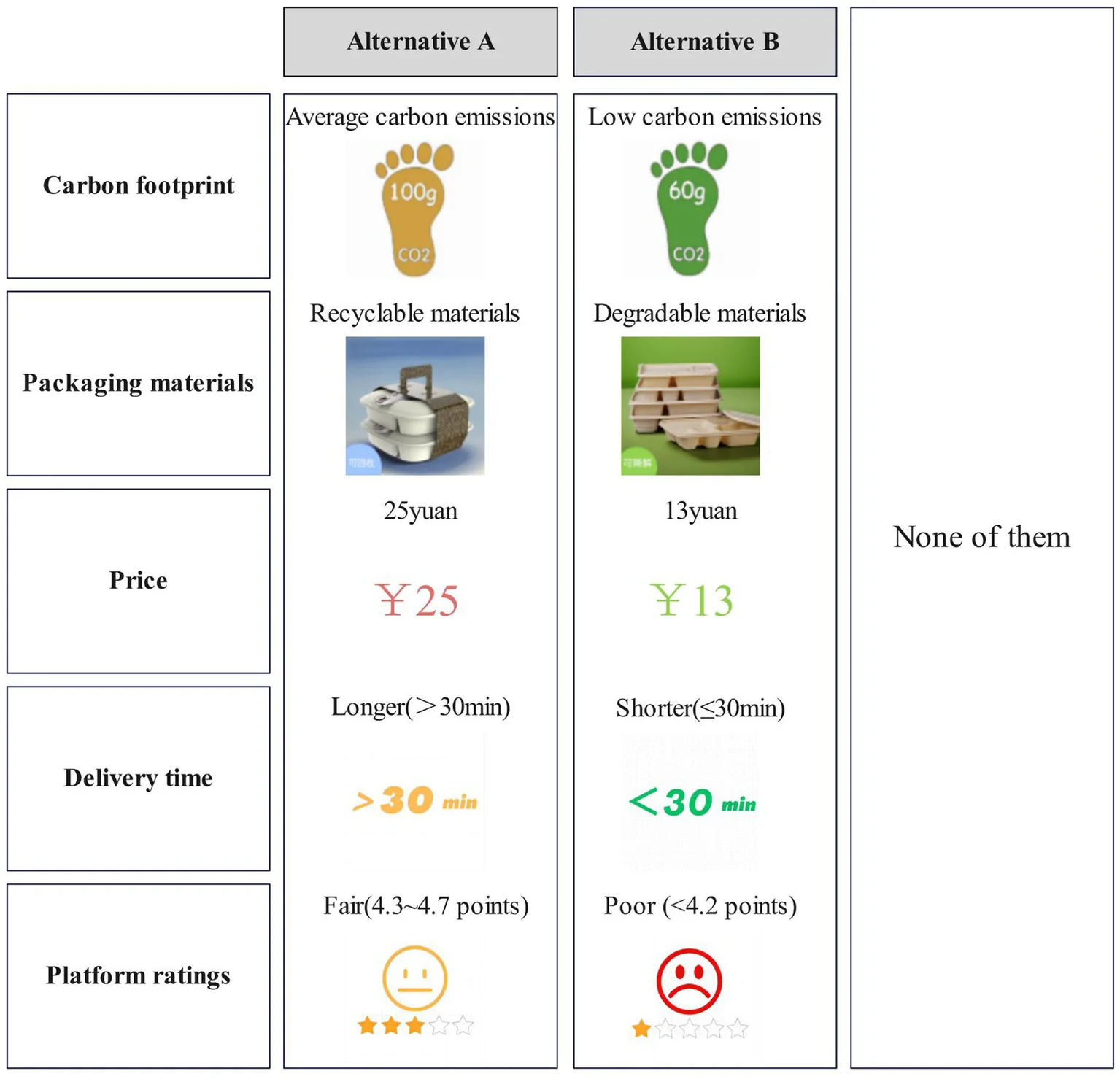
Example of choice tasks.

In the takeout choice experiment section, given the relatively limited consumer familiarity with carbon labels documented in previous Chinese studies (58), this study briefly introduces the concept of carbon footprint to consumers through relevant explanatory information before conducting the choice experiment to ensure that consumers understand the meaning of carbon labels. For a takeout food item, its carbon footprint refers to the total carbon emissions from ingredient preparation, packaging, delivery transportation, and waste disposal.

In the discrete choice model, the variables involved in studying consumer preferences for takeout food with carbon labels and their willingness to pay are the specific level variables of the takeout food attributes (Table 2). Apart from price and the exit option (choosing nothing, named as None), the rest of the variables are encoded using dummy variables.

**Table 2:** Description and definition of attribute variables.

Attribute category	Attribute	Variables of level	Variable Definition
Traditional attributes	Price	Price	Continuous variable, exit = 0
	Platform ratings	Poor* Fair Excellent	Yes = 1, no request or exit = 0 Yes = 1, no request or exit = 0 Yes = 1, no request or exit = 0
	Delivery time	Shorter Longer*	Yes = 1, no request or exit = 0 Yes = 1, no request or exit = 0
Carbon-related attributes	Carbon footprint	Low-CF Average-CF High-CF*	Yes = 1, no request or exit = 0 Yes = 1, no request or exit = 0 Yes = 1, no request or exit = 0
	Packaging materials	Disposable* Recyclable Degradable	Yes = 1, no request or exit = 0 Yes = 1, no request or exit = 0 Yes = 1, no request or exit = 0

Variables marked with an asterisk (*) will be treated as reference variables.

### 3.3 Theoretical model

In this study, a discrete choice model is constructed to describe consumers’ choices when hypothetically purchasing takeout food. The discrete choice experiment is grounded in Lancaster's (70) characteristics theory of consumer demand and random utility theory (71, 72). Under this framework, respondents evaluate alternatives described by bundles of attributes and choose the one that yields the highest utility (73). Two features make this approach particularly suitable for the present study. First, it allows estimation of marginal valuations for individual attributes and the trade-offs among them, enabling preferences for carbon-related attributes to be compared directly with those for conventional service attributes rather than examined in isolation. Second, because carbon labels have not yet been widely implemented in China’s online food-delivery market, a stated-preference approach is particularly appropriate, as revealed-preference data on carbon-labelled orders are not yet available.

Random utility theory assumes that each alternative provides a specific level of utility to the decision-maker (74). For consumer n, alternative j in choice set t provides utility $U_{\mathrm{njt}}$ , which consists of a systematic component determined by the observable attributes of the alternative and a random component capturing unobserved influences (75). Accordingly, the utility obtained by consumer n from alternative j in choice set t is expressed as:


$$U_{\mathrm{njt}}=V_{\mathrm{njt}}+\varepsilon_{\mathrm{njt}}$$ (1)

$$V_{\mathrm{njt}}=\beta_{n}x_{\mathrm{njt}}$$ (2)

In Equation 1, utility U is composed of fixed utility $V_{\mathrm{njt}}$ and a random term $\varepsilon_{\mathrm{njt}}$ . In Equation 2, β represents the utility score vector, indicating an individual’s preferences; $x_{\mathrm{njt}}$ is the attribute vector of alternative j chosen by consumer n under choice set *t*. According to utility maximization theory, subjects always choose the alternative that offers them the greatest utility. Therefore, the probability of subject *n* choosing alternative from *J* alternatives under choice set t is given in Equation 3:


$$P_{\mathrm{njt}}=\text{prob}(y_{\mathrm{njt}}=j)=\text{prob}(U_{\mathrm{njt}}-U_{\mathrm{nlt}}>0)\;\forall l\neq j$$ (3)

### 3.4 Empirical model

In discrete choice analysis, the conditional Logit model, mixed Logit model, and latent class model are the main model types. The conditional Logit model is suitable for analyzing the impact of attributes on individual choice behavior; the mixed Logit model is more flexible in dealing with inter-individual preference heterogeneity. The conditional Logit and mixed Logit models were estimated using Stata 18.0 to examine consumers’ preferences and willingness to pay for carbon-labeled takeout food. At the same time, interaction terms were further incorporated into the conditional Logit model to explore the sources of preference heterogeneity. The latent class model is further used to represent preference heterogeneity as a discrete distribution and to identify consumer segments with distinct patterns of food-delivery preferences.

By assuming that the random term $\varepsilon_{\mathrm{njt}}$ follows an independent and identically distributed distribution, and follows an Extreme Value Type I (Gumbel) distribution, then the model can be estimated through the conditional logit model. Equation 4 shows the basic specification of the conditional logit model.


$$P_{\mathrm{njt}}=\frac{\exp(\beta_{n}x_{\mathrm{njt}})}{\sum_{j=1}^{J}\exp(\beta_{n}x_{\mathrm{njt}})}$$ (4)

The mixed Logit model, also known as the random parameter Logit model, is highly flexible, allowing the coefficients of explanatory variables to vary randomly among subjects, meaning the coefficient values follow a probability distribution. This model can estimate any form of mixed distribution and assumes the distribution of coefficients before estimation, enabling the verification of the existence of preference heterogeneity among subjects. In this model, the probability of consumer n choosing alternative i in choice set t is determined by the probability density function of the coefficients (as shown in Equation 5), and the estimable parameters of this density function can provide important information about preference heterogeneity (76). In Equation 5, $f(\beta_{n}|\theta )$ is the probability density function of coefficient $\beta_{n}$ , and θ is the estimable parameters of this density function.


$$P(n,i,t)=\int \frac{\exp(\beta_{n}x_{\mathrm{nit}})}{\sum_{j=1}^{J}\exp(\beta_{n}x_{\mathrm{njt}})}f(\beta_{n}|\theta )d\beta_{n}$$ (5)

The latent class model (LCM) represents preference heterogeneity as a discrete distribution across a finite number of latent classes. In contrast, the mixed logit model allows preference parameters to vary continuously across individuals, whereas the LCM assumes preference parameters are homogeneous within each class but differ across classes (77). This specification makes it possible to identify consumer segments exhibiting distinct trade-offs among the attributes included in the choice experiment.

In this study, the LCM comprises two components, with distinct variables entering each component. Within each class, the class-specific utility function includes eight variables derived from the attributes in Table 2: price, excellent and fair platform ratings, shorter delivery time, degradable and recyclable packaging, and low- and average-carbon-footprint levels. Price enters the model as a continuous variable, while the remaining seven variables are dummy coded, with poor platform rating, longer delivery time, disposable packaging, and high carbon footprint serving as the respective reference levels. The class-membership model includes five individual-level covariates: gender, age, monthly living expenses, subjective environmental knowledge, and environmental attitude. These covariates do not enter the class-specific utility function; instead, they are used to model the probability that a respondent belongs to a given latent class. Class 4 serves as the reference class in the class-membership model, so the class-membership coefficients for Classes 1–3 are estimated relative to Class 4. Models with different numbers of classes were estimated, and the preferred specification was selected by comparing the Akaike Information Criterion (AIC), Bayesian Information Criterion (BIC), and Consistent Akaike Information Criterion (CAIC) (78). Assuming that subject n belongs to category c, the probability of them choosing option *i* in choice set t (out of T total choice sets) is given in Equation 6 as follows:


$$P(n,|i,|t,|c)=\prod_{t=1}^{T}\frac{\exp(\beta_{c}x_{\mathrm{njt}})}{\sum_{j=1}^{J}\exp(\beta_{c}x_{\mathrm{njt}})}$$ (6)

Additionally, this paper will calculate the willingness to pay (WTP), reflecting the amount a subject is willing to pay extra for a particular feature of a product compared to a reference class, with the expression given in Equation 7 as follows (79):


$$\mathrm{WTP}=-\frac{\beta_{n}}{\beta_{\text{price}}}$$ (7)

As mentioned above, WTP is commonly represented by the ratio of the coefficients of the attribute variables to the coefficients of the payment price variable. Moreover, WTP is a relative value; interpreting this number directly is meaningless, and it only makes sense to interpret it through the switching of attributes. At the same time, this paper calculates the confidence interval of the willingness to pay using the delta method, first calculating the variance of the willingness to pay based on Equation 8, with the expression as follows:


$$\mathrm{var}(\mathrm{WT}P_{n})=[\begin{array}{l} {\left(-\frac{1}{\beta_{p\text{rice}}}\right)}^{2}\mathrm{var}(\beta_{n})+{\left(\frac{\beta_{n}}{\beta_{\text{price}}^{2}}\right)}^{2}\mathrm{var}(\beta_{\text{price}})+\\ 2(-\frac{1}{\beta_{\text{price}}})(\frac{\beta_{n}}{\beta_{\text{price}}^{2}})\mathrm{cov}(\beta_{n},\beta_{\text{price}}) \end{array}]$$ (8)

Next, calculate the confidence interval of the willingness to pay using Equation 9, as follows:


$$95\%\mathrm{CI}=\mathrm{WT}P_{n}\pm Z_{a/2}\sqrt{\mathrm{var}(\mathrm{WT}A_{n}}$$ (9)

## 4 Results

### 4.1 Descriptive analysis of demographics

Among the 692 valid responses, 427 respondents completed questionnaire version A and 265 completed questionnaire version B. Since each respondent made six choices, this yields 4,152 choice observations and, with three alternatives per choice set, 12,456 observations for estimation. In terms of demographics, the average age of students participating in this survey was 21.1 years, with males accounting for 57.9% and females 42.1%. The students hailed from various disciplines, with the highest proportion being economics students (24%), followed by science (12%) and management (12%). Regarding takeout consumption habits, as illustrated in Figure 2, 55.8% of respondents reported ordering takeout three to seven times per week, while 44.2% did so more than seven times per week. Thus, all respondents were high-frequency users who ordered takeout at least three times per week. As for monthly living expenses, 33.4% of students reported spending between ¥1,000 and ¥1,500 per month, while 44.7% spent between ¥1,500 and ¥2,000. Moreover, concerning the proportion of staple food in takeout consumption, 48.3% of students indicated that staple food accounted for 40 to 60% of their total takeout expenditure, while 33.4% were between 60 to 80%. Detailed descriptive statistics are reported in Table 3.

**Figure 2:**
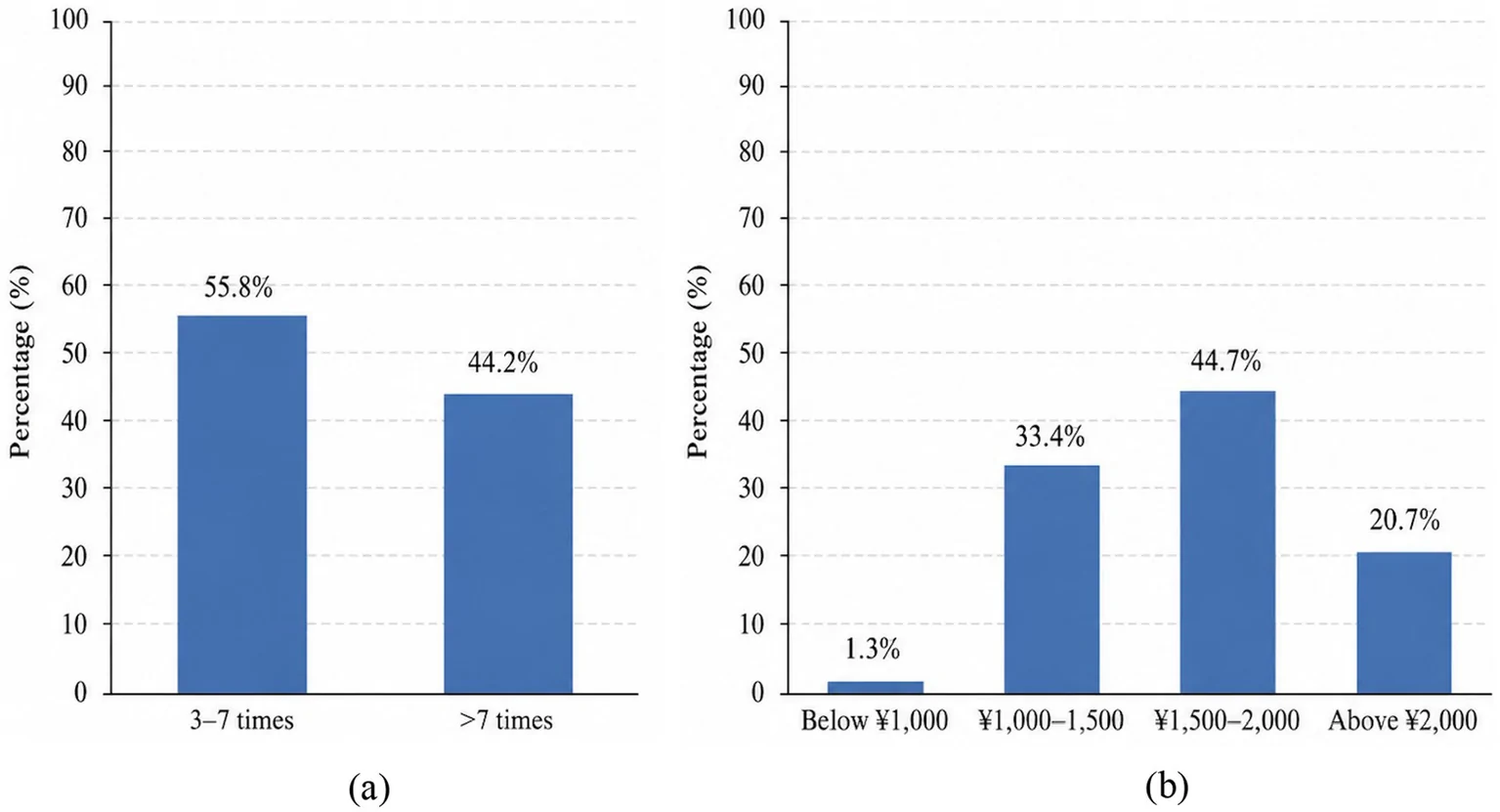
Key consumption characteristics of the survey respondents. (a) Weekly frequency of online food-delivery consumption. (b) Monthly living expenses.

**Table 3:** Descriptive characteristics of the sample.

Personal characteristics	Proportion (%)	Personal characteristics	Proportion (%)
Gender		Major	
Male	57.9	Economics	24
Female	42.1	Science	12
Weekly frequency of takeaway consumption		Management	12
3–7 times	55.8	Literature	10.5
More than 7 times	44.2	Engineering	9.5
Monthly living expenses		History	6.8
Below 1,000 yuan	1.3	Agriculture	6.6
1,000 yuan to 1,500 yuan	33.4	Law	5.2
1,500 yuan to 2000 yuan	44.7	Philosophy	4.6
Above 2000 yuan	20.7	Education	4.1
Proportion of staple food in takeaway consumption		Art	2.8
40% ~ 60%	48.3	Medicine	1.6
60% ~ 80%	33.4	Military Studies	0.3
Above 80%	18.4		

In terms of subjects’ subjective perceptions, previously through the fourth part of the questionnaire survey, this work quantitatively evaluated participants’ “environmental attitudes” and “subjective environmental knowledge” using a 5-point Likert scale and a 7-point Likert scale, respectively. Both sections comprised 15 questions. Table 4 presents the scoring results: participants scored an average of 3.779 (out of a possible 5) on environmental attitudes, with a standard deviation of 0.689; for subjective environmental knowledge, utilizing a 7-point scale, the average score was 4.693 (out of a possible 7), above the midpoint, with a standard deviation of 1.219. These data reflect a generally positive attitude towards environmental issues and a higher level of self-perceived environmental knowledge among participants, while also revealing some degree of individual variability in these two dimensions.

**Table 4:** Consumers’ subjective perception situation.

Situation category	Mean	Std.	Max	Min
Environmental attitude	3.779	0.689	5	1.733
Subjective environmental knowledge	4.693	1.219	7	1.133

### 4.2 The choice preference analysis

Tables 5, 6 respectively display the estimation results of the conditional Logit model and mixed Logit model, where the mean coefficients reflect the overall preference direction relative to the reference level: positive coefficients indicate a general preference for that attribute scheme, while negative coefficients represent a general dispreference. According to Tables 5, 6, the results of the conditional Logit and mixed Logit are generally consistent, and the following analysis will focus on the estimation results of the mixed Logit.

**Table 5:** Estimation results of conditional logit model.

Attribute	Coefficient	Coeff. Std.
Price	−0.034^***^	0.004
Excellent	0.637^***^	0.053
Fair	0.178^***^	0.053
Shorter	−0.024	0.037
Degradable	0.265^***^	0.052
Recyclable	0.127^**^	0.050
Low-CF	0.449^***^	0.049
Average-CF	0.420^***^	0.052
None	−2.176^***^	0.116
Observations	12,456	
Log likelihood	−3384.5632	
Prob > chi^2^	0.0000	

***, **, and * indicate 1, 5, and 10% significance levels, respectively. The same applies below.

**Table 6:** Mixed logit estimates.

Attribute	Coefficient mean		Coefficient standard deviation	
	Estimate	Standard error	Estimate	Standard error
Price	−0.049^***^	0.006		
Excellent	0.893^***^	0.100	1.140^***^	0.117
Fair	0.238^***^	0.093	0.381^**^	0.175
Shorter	−0.022	0.069	0.567^***^	0.109
Degradable	0.353^***^	0.078	0.842^***^	0.132
Recyclable	0.139^**^	0.062	0.040	0.347
Low-CF	0.574^***^	0.085	1.180^***^	0.128
Average-CF	0.553^***^	0.072	0.759^***^	0.132
None	−2.353^***^	0.134		
Observations	12,456			
Log likelihood	−3308.9519			
Prob>chi^2^	0.0000			

When evaluating traditional attributes of takeout food, the estimated coefficient for excellent platform ratings is significantly positive (coefficient = 0.893, *p* < 0.01), indicating that consumers have clear expectations and inclinations towards service quality when choosing takeout. However, the significant standard deviations for platform rating (SD = 1.140, *p* < 0.01) and delivery time (SD = 0.567, *p* < 0.01) indicate substantial heterogeneity in respondents’ preferences for these conventional service attributes. In terms of price sensitivity, consumers show a general concern for cost-effectiveness, reflected in the preference for lower-priced takeout options, with the negative coefficient (coefficient = −0.049, *p* < 0.01) strongly pointing to a consumption pattern dominated by cost–benefit. Additionally, when facing takeout food labeled with different carbon footprint levels, consumers show a significantly positive preference for low (coefficient = 0.574, *p* < 0.01) and medium (coefficient = 0.553, *p* < 0.01) carbon footprint products, preferring low and medium carbon footprint options over high carbon footprint choices. The significant standard deviation of low (SD = 1.180, *p* < 0.01) and medium (SD = 0.759, *p* < 0.01) carbon footprints also highlights the individual heterogeneity in environmental attribute choices. In terms of meal box packaging materials, compared to disposable packaging, consumers show a significant preference for green packaging options (degradable materials, recyclable materials), and compared to recyclable materials, degradable materials are more favored by consumers.

Based on the estimation results in Table 6, the willingness to pay (WTP) for traditional and carbon-related attributes of takeout food (Table 7 and Figure 3) was calculated to reflect the emphasis on these attributes. The results show that the platform rating of takeout food, as an important reference index for product quality, has a significant and profound impact on consumer decision-making. Excellent platform ratings command the largest premium, followed by low- and medium-carbon-footprint levels. Both degradable and recyclable packaging also have positive WTP estimates, whereas the confidence interval for shorter delivery time includes zero.

**Table 7:** Consumers’ WTP for food-delivery attributes.

Attribute	WTP	95% Confidence Interval
Excellent	18.29^***^	[13.63,22.94]
Fair	4.87^***^	[2.05,7.68]
Shorter	−0.45	[−2.79,1.89]
Degradable	7.22^***^	[3.81,10.63]
Recyclable	2.85^**^	[0.16,5.55]
Low-CF	11.77^***^	[7.25,16.29]
Average-CF	11.33^***^	[7.66,14.99]

**Figure 3:**
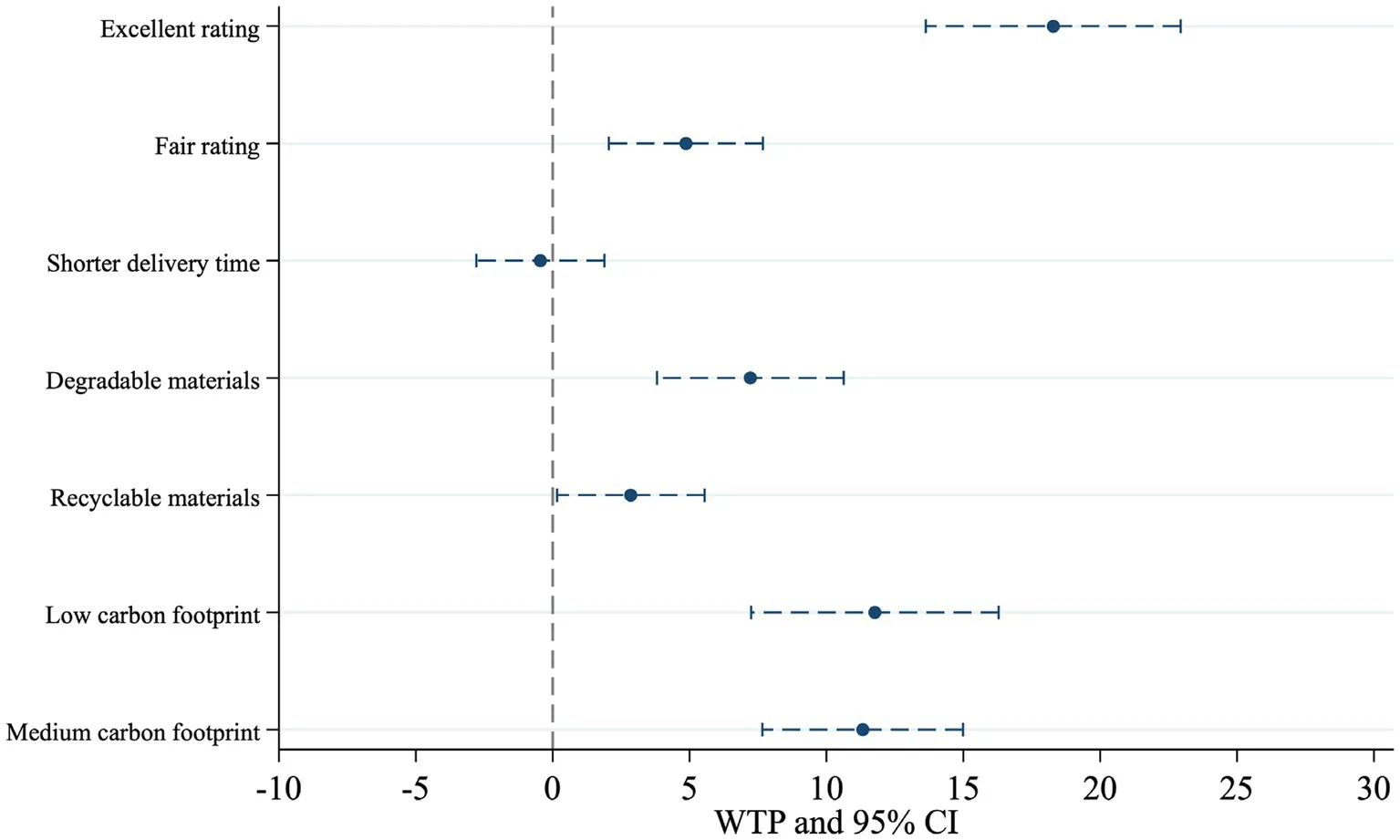
Estimated WTP for online food-delivery attributes. Points represent WTP estimates in yuan per order, and horizontal bars indicate 95% confidence intervals.

Specifically, under the same conditions, compared to services with lower ratings, consumers show higher recognition for highly rated takeout services (95% CI: 13.63–22.94, *p* < 0.01) and are willing to pay an extra 18.29 yuan/meal (approximately $2.56 USD/meal) for a higher quality and more satisfactory service experience. Regarding the carbon footprint, the study results indicate that consumers are willing to pay an additional 11.77 yuan/meal (approximately $1.65 USD/meal) for low-carbon-footprint takeout food (95% CI: 7.25–16.29, *p* < 0.01), which is equivalent to 61.9% of the mean price level used in the choice experiment. And the willingness to pay for medium-carbon-footprint products (95% CI: 7.66–14.99, *p* < 0.01) is 11.33 yuan/meal (approximately $1.59 USD/meal), which is equivalent to 59.6% of the mean price level. The close values of these two may reflect the relatively weak recognition and sensitivity of consumers to specific carbon footprint levels. In terms of takeout packaging material choices, consumers show a clear preference for environmentally friendly materials. Especially for degradable materials, compared to disposable packaging materials (95% CI: 3.81, 10.63, *p* < 0.01), consumers are on average willing to pay an additional 7.22 yuan/meal (approximately $1.01 USD/meal). This result reflects the growing public awareness of environmental protection and the high importance placed on the use of sustainable materials. The preference for recyclable materials (95% CI: 0.16–5.55, *p* < 0.05) is also reflected, although the willingness to pay (2.85 yuan/meal, approximately $0.40 USD/meal) is lower than for degradable materials, it still indicates a positive attitude towards environmentally friendly packaging.

### 4.3 Heterogeneity analysis

The estimation results of the mixed Logit model reveal significant preference heterogeneity among consumers for takeout food with carbon labels. This finding prompts us to further explore the deep factors causing consumer preference heterogeneity. In further analysis, this work plans to use the conditional Logit model as the research framework to discuss the interaction effects of carbon-related attributes (including carbon footprint and meal box packaging material) with socio-economic characteristics (age, gender, and monthly living expenses), environmental attitudes, and subjective environmental knowledge.

Before model estimation, the scale score data were centered and standardized, followed by the KMO test and Bartlett’s test of sphericity. The test results show that the KMO values for environmental attitudes and environmental knowledge scales reached 0.933 and 0.95, respectively, and the Bartlett test values were significant, proving that the data are suitable for principal component analysis. Therefore, this study extracted the main components and calculated the composite scores for “environmental attitudes” and “subjective environmental knowledge.” To accurately assess the interactions among these variables, interaction terms were included in two conditional logit models (CLMs), and terms with *p*-values below 0.2 were retained using backward elimination to ensure the models’ statistical significance and explanatory power. The final regression analysis results are shown in Table 8, detailing the specific regression results of CLM A (including carbon footprint interaction terms) and CLM B (including meal box packaging material interaction terms).

**Table 8:** The impact of consumer heterogeneity on their preferences.

Category		CLM A		CLM B	
		Coefficient	Coeff. Std.	Coefficient	Coeff. Std.
Attribute					
	Price	−0.0339^***^	0.00373	−0.0354^***^	0.00378
	Excellent	0.648^***^	0.0538	0.631^***^	0.0535
	Fair	0.186^***^	0.0531	0.177^***^	0.0528
	Shorter	−0.0132	0.0379	−0.0160	0.0377
	Degradable	0.272^***^	0.0525	−0.383	0.444
	Recyclable	0.126^**^	0.0502	0.124^**^	0.0498
	Low-CF	−0.0476	0.187	0.437^***^	0.0496
	Average-CF	−0.550	0.464	0.419^***^	0.0521
	None	−2.163^***^	0.117	−2.208^***^	0.117
Socio-economic variables					
	Interacting with carbon footprint				
	Low-CF × Gender	0.389^***^	0.0939	–	–
	Low-CF × EA	0.113^***^	0.0269	–	–
	Low-CF × S-EK	0.0640^***^	0.0188	–	–
	Low-CF × M-LE	0.0985	0.0599	–	–
	Average-CF × Age	0.0268	0.0203	–	–
	Average-CF × Gender	0.239^***^	0.0904	–	–
	Average-CF × M-LE	0.0968^*^	0.0588	–	–
	Interacting with food packaging materials				
	Degradable × Age	–	–	0.0307	0.0210
	Degradable × S-EK	–	–	0.0553^***^	0.0213
	Degradable × EA	–	–	−0.0388	0.0263
	Recyclable × S-EK	–	–	0.0567^***^	0.0201

In this table, EA, S-EK, M-LE are abbreviations for Environmental Attitude, Subjective Environmental Knowledge, Monthly Living Expenses, respectively. ***, **, and * indicate 1, 5, and 10% significance levels, respectively.

The analysis results of CLM A reveal significant positive interactions between gender (coefficient = 0.389, *p* < 0.01), environmental attitudes (coefficient = 0.113, *p* < 0.01), and subjective environmental knowledge (coefficient = 0.064, *p* < 0.01) with low carbon footprint, indicating that males with higher environmental consciousness and knowledge levels prefer to choose takeout food with a low carbon footprint. Consumers with more positive environmental attitudes or deeper environmental knowledge tend to choose takeout food with a low carbon footprint. At the same time, the preference for medium carbon footprint takeout food is significantly positively correlated with gender (coefficient = 0.239, *p* < 0.01) and monthly living expenses (coefficient = 0.0968, *p* < 0.1), indicating that males with higher monthly living expenses prefer to choose takeout food with a medium carbon footprint, reflecting the influence of income level on consumer environmental behavior choices. In CLM B, although the choice of degradable materials does not show a significant interaction with age and environmental attitudes, the positive interaction with subjective environmental knowledge (coefficient = 0.0553, *p* < 0.01) is significant, indicating that consumers with a deeper understanding of environmental issues prefer to choose meal boxes made of degradable materials. Similarly, the significant positive interaction between the choice of recyclable materials and subjective environmental knowledge (coefficient = 0.0567, *p* < 0.01) further confirms the positive impact of environmental knowledge on promoting the choice of environmentally friendly packaging, but the interaction between environmental attitudes and eco-friendly packaging materials is not significant, suggesting that strengthening environmental attitudes does not significantly promote consumers’ choice of eco-friendly packaging.

### 4.4 Latent model analysis

#### 4.4.1 Determining the number of latent classes

Table 9 reports the fit statistics for latent class models containing two to eight classes. Although AIC decreases as the number of classes increases and reaches its minimum for the eight-class specification, both BIC and CAIC are minimized for the four-class model. The four-class specification was therefore selected for the subsequent analysis.

**Table 9:** LCM fit statistics.

Classes	LLF	AIC	CAIC	BIC
2	−3272.359	6582.718	6687.97	6668.97
3	−3235.034	6528.068	6688.716	6659.716
4	−3170.409	6418.818	6634.862	6595.862
5	−3153.444	6404.887	6676.327	6627.327
6	−3132.729	6383.459	6710.295	6651.295
7	−3109.666	6357.332	6739.563	6670.563
8	−3088.404	6334.808	6772.435	6693.435

#### 4.4.2 Results of the LCM

The latent class model identifies four distinct preference segments (Table 10) to further examine heterogeneity in consumers’ online food-delivery preferences. As shown in Figure 4, the Affordable and Eco-friendly class is the largest, accounting for 51.5% of the sample, followed by the Carbon-Sensitive class (22.3%), the Cost-Performance Priority class (19.0%), and the Low-Carbon Consumption class (7.2%). Class 4 serves as the reference class for the class-membership model. In addition, age enters the class-membership model as a continuous covariate. Therefore, a positive age coefficient for Classes 1–3 indicates that, as age increases, respondents are relatively more likely to belong to the corresponding class than to Class 4.

**Table 10:** Four-class latent class model estimates.

Attribute	Class 1 (carbon-sensitive)		Class 2 (Cost-Performance Priority)		Class 3 (Affordable and Eco-friendly)		Class4 (Low Carbon Consumption)	
	Coef.	Std.error	Coef.	Std.error	Coef.	Std.error	Coef.	Std.error
Price	0.013	0.014	−0.239^***^	0.020	−0.028^***^	0.006	−0.068^***^	0.023
Excellent	1.712^***^	0.325	5.173^***^	0.493	0.064	0.095	0.787^*^	0.444
Fair	0.273	0.227	1.142^***^	0.283	0.080	0.083	0.506	0.367
Shorter	−0.527^**^	0.238	−0.686^**^	0.268	0.192^***^	0.065	0.135	0.219
Degradable	1.901^***^	0.362	0.148	0.237	0.289^***^	0.082	0.545	0.391
Recyclable	1.290^***^	0.264	−0.382	0.266	0.110	0.077	0.136	0.349
Low-CF	4.423^***^	0.511	−1.085^***^	0.263	−0.009	0.091	1.179^***^	0.382
Average-CF	2.106^***^	0.310	0.762	0.493	0.444^***^	0.085	0.564	0.407
Gender	0.755^*^	0.397	−0.215	0.416	0.139	0.376		
Age	0.333^***^	0.047	0.146^*^	0.077	0.307^***^	0.050		
M-LE	0.277	0.203	0.086	0.220	0.126	0.185		
S-EK	0.284^***^	0.105	−0.078	0.095	0.082	0.090		
EA	−0.124	0.144	0.015	0.147	−0.561^***^	0.134		
Proportion	22.3%		19%		51.5%		7.2%	

In this table, EA, S-EK, M-LE are abbreviations for Environmental Attitude, Subjective Environmental Knowledge, Monthly Living Expenses, respectively.

**Figure 4:**
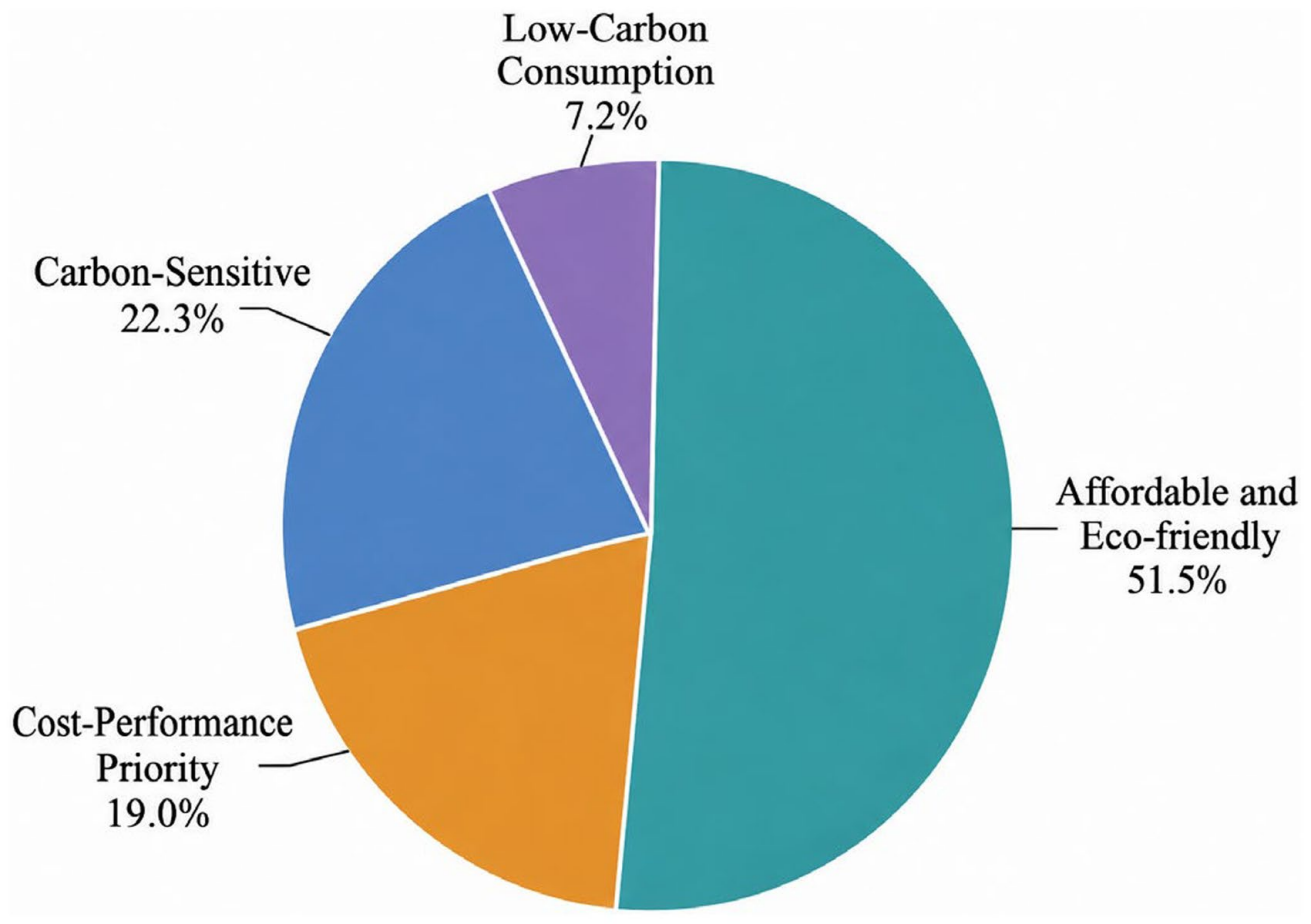
Relative sizes of the four latent classes.

Class 1—Carbon-Sensitive Type (22.3%). This group exhibits the strongest preferences for carbon-related attributes. Both low- (coefficient = 4.423, *p* < 0.01) and medium-carbon-footprint options (coefficient = 2.106, *p* < 0.01) are positively valued, with the coefficient for low carbon footprint being particularly large. Degradable and recyclable packaging are also positively preferred, while excellent platform ratings remain important. Relative to Class 4, membership in this class is positively associated with age (coefficient = 0.333, *p* < 0.01), being male (coefficient = 0.755, *p* < 0.1), and higher subjective environmental knowledge (coefficient = 0.284, *p* < 0.01).

Class 2—Cost-Performance Priority Type (19.0%). Consumers in this class are highly price-sensitive (coefficient = −0.239, *p* < 0.01) and place particularly strong weight on excellent platform ratings (coefficient = 5.173, *p* < 0.01), indicating that price and service quality dominate their choices. In contrast, the coefficient for low carbon footprint is significantly negative (coefficient = −1.085, *p* < 0.01), while neither packaging option is significantly preferred, indicating they are less willing to pay extra for measures to reduce carbon emissions and focus more on the quality of the traditional consumption experience. Relative to Class 4, membership in this class is positively associated with age (coefficient = 0.146, *p* < 0.1).

Class 3—Affordable and Eco-friendly Type (51.5%). As the largest segment, this group combines price sensitivity (coefficient = −0.028, *p* < 0.01) with selective preferences for environmental attributes (coefficient = −0.561, *p* < 0.01), but platform ratings (coefficient = 0.064, *p* > 0.1) do not significantly affect their choices. Consumers significantly prefer shorter delivery times (coefficient = 0.192, *p* < 0.01) and degradable packaging (coefficient = 0.289, *p* < 0.01), and they positively value medium-carbon-footprint options (coefficient = 0.444, *p* < 0.01), whereas the coefficient for low carbon footprint is not significant (coefficient = −0.009, *p* > 0.1). Relative to Class 4, membership in this class is associated with older age (coefficient = 0.307, *p* < 0.01) and less positive environmental attitudes (coefficient = −0.561, *p* < 0.01).

Class 4—Low-Carbon Consumption Type (7.2%). This group is price-sensitive (coefficient = −0.068, *p* < 0.01) but exhibits a clear preference for low-carbon-footprint options (coefficient = 1.179, *p* < 0.01). By contrast, the coefficients for degradable (coefficient = 0.545, *p* > 0.1) and recyclable packaging (coefficient = 0.136, *p* > 0.1) are positive but statistically insignificant, and most conventional service attributes are also insignificant except for a weak preference for excellent platform ratings (coefficient = 0.787, *p* < 0.1).

Taken together, the latent class model results further indicate that sociodemographic characteristics and environmental cognition contribute to preference heterogeneity across consumer segments. Higher age is positively associated with membership in the Carbon-Sensitive, Cost-Performance Priority, and Affordable and Eco-friendly classes relative to the Low-Carbon Consumption class, while male respondents and those with higher subjective environmental knowledge are more likely to belong to the Carbon-Sensitive class. Environmental attitudes also differentiate the Affordable and Eco-friendly class from the reference group. These patterns suggest that age, gender, subjective environmental knowledge, and environmental attitudes help explain the observed segmentation of food-delivery preferences.

## 5 Discussion

### 5.1 Preferences and WTP for carbon-related attributes

The results show that college students derive significantly greater utility from food-delivery options with low or medium carbon footprints than from those with a high carbon footprint. This finding is consistent with experimental evidence that salient information about carbon can redirect consumers towards lower-emission food choices (52, 53, 80). The preference for low- and medium-carbon-footprint options suggests that respondents may respond more strongly to avoiding an explicitly high-carbon alternative than to finer distinctions between lower emission levels (55). Moreover, the WTP for an excellent platform rating exceeds that for the carbon-related attributes, indicating that environmental information matters but does not displace conventional concerns about service quality. Students also prefer degradable and recyclable packaging to disposable packaging, with a higher estimated WTP for degradable packaging. This difference may reflect its more readily perceived environmental benefits, although this valuation captures consumers’ perceptions rather than the objective lifecycle performance of the packaging alternatives (81, 82).

From a behavioral-economics perspective, carbon labels can be viewed as informational nudges that increase the salience of otherwise difficult-to-observe environmental consequences without restricting consumers’ choices or directly changing relative prices. This mechanism differs from the default and green-point intervention examined by He et al. (18), which substantially increased orders without disposable cutlery by altering the digital choice architecture. The present findings indicate that making carbon footprints and packaging materials visible may similarly direct attention towards environmental consequences at the point of purchase. However, carbon labels should be regarded as potentially low-cost and context-dependent nudges rather than uniformly effective interventions. Existing field evidence shows that their effects depend on label format, comprehensibility, placement, and repeated exposure (53, 54). Carbon labels may therefore be most effective when combined with clear explanations, prominent positioning, and other transparent platform-based measures. Discounts, coupons, and reward points should be treated as complementary economic incentives rather than pure nudges because they directly alter the material consequences of consumers’ choices.

### 5.2 Environmental knowledge and the attitude-behavior relationship

Subjective environmental knowledge strengthens preferences for both lower-carbon-footprint options and environmentally friendly packaging, whereas pro-environmental attitudes are significantly associated only with preferences for lower-carbon-footprint options. Besides, the link between environmental attitudes and the choice of eco-friendly packaging is not significant. This asymmetry suggests that general environmental concern may be sufficient for consumers to respond to an intuitive traffic-light carbon label. Still, packaging choices require a more specific understanding of degradability, recyclability, and waste treatment. The result is consistent with evidence that subjective knowledge can directly shape sustainable consumption and that uncertainty about packaging materials may impede environmentally friendly choices (83, 84). It also helps explain why environmental attitudes do not always translate into consistent behavior across product attributes. Ketelsen et al. (85) found that consumers face challenges in identifying eco-friendly packaging and understanding new materials like bio-based packaging. Despite generally positive attitudes towards eco-friendly packaging, a lack of knowledge and awareness may prevent these attitudes from translating into action. Carbon-related disclosure should therefore be designed not only to attract attention but also to help consumers interpret the environmental meaning of the information presented. Repeated exposure and opportunities for reflection may further strengthen these effects, as suggested by Casati et al. (54) and Banerjee et al. (86).

### 5.3 Preference heterogeneity and differentiated interventions

The mixed logit and latent class results demonstrate that average preferences conceal substantial heterogeneity in consumers’ trade-offs among carbon information, packaging materials, price, platform ratings, and delivery time. Specifically, the LCM identifies four distinct segments: Carbon-Sensitive consumers, who place strong value on lower-carbon options and environmentally friendly packaging; Cost-Performance Priority consumers, whose choices are dominated by price and platform ratings; Affordable and Eco-friendly consumers, the largest segment, who remain price-sensitive while selectively valuing degradable packaging and average-carbon-footprint options; and Low-Carbon Consumption consumers, who show a clear preference for low-carbon-footprint options but relatively limited responses to packaging and most conventional service attributes. These distinct preference profiles suggest that uniform interventions are unlikely to be equally effective across consumers, consistent with recent evidence of substantial heterogeneity in responses to sustainability labels and food-policy interventions (87, 88). Carbon-sensitive consumers may respond more strongly to salient carbon information and repeated environmental feedback. In contrast, consumers balancing affordability and environmental performance may require carbon labels to be complemented by modest price incentives. Cost-performance-oriented consumers may be more receptive when lower-carbon options retain reliable platform ratings and service-quality assurances, particularly because conventional attributes such as price and quality remain important determinants of food choices.

### 5.4 Limitations

Nevertheless, several limitations of this study should be acknowledged. First, the WTP estimates are derived from hypothetical choice scenarios rather than observed market transactions. As with other stated-preference studies, they may therefore be subject to hypothetical bias, while environmentally desirable attributes may also be susceptible to social-desirability concerns. The estimated premiums should consequently be interpreted as stated valuations of the relative importance respondents attach to different food-delivery attributes rather than as direct predictions of the premiums they would necessarily pay in actual market settings.

Second, the external validity of the results is bounded by the study population. The sample consists of university students in the Sichuan-Chongqing region, a group deliberately selected because of its intensive use of online food-delivery services. Nevertheless, the magnitude of the estimated preferences and WTP may differ across age groups, regions, and consumer populations with different socioeconomic and environmental characteristics. The findings should therefore be interpreted primarily as evidence for frequent young urban users in comparable inland metropolitan settings.

## 6 Conclusion and policy implications

### 6.1 Conclusion

Understanding whether environmental information can translate into lower-carbon food choices is increasingly important as online food delivery becomes a major component of urban consumption and raises growing concerns about carbon emissions and packaging waste. Accordingly, the study examines how carbon-footprint disclosure and packaging information influences college students’ online food-delivery choices and quantifies their willingness to pay for these environmental attributes. Using a discrete choice experiment with 692 college students in the Sichuan-Chongqing region and applying conditional logit, mixed logit, and latent class models, the analysis further investigates preference heterogeneity across consumers.

Based on the empirical results, three main conclusions can be drawn: (1) Carbon labels disclosure may be helpful to encourage Chinese university students to choose low-carbon food delivery options, with these consumers willing to pay a premium for food with a lower carbon footprint or more sustainable packaging (e.g., biodegradable or recyclable materials). (2) Males and individuals with a positive environmental attitude and higher levels of environmental knowledge are more likely to choose low-carbon footprint products, while higher-income males tend to opt for medium-carbon footprint food delivery options. Additionally, increased environmental knowledge encourages more consumers to select green packaging. However, the link between environmental attitudes and the choice of eco-friendly packaging is not significant. (3) Consumers can be categorized into four distinct groups, each demonstrating varying preferences in terms of carbon sensitivity, value for money, and environmental responsibility. This diversity suggests that tailored strategies targeting these different segments could be more effective. Notably, more than half of the consumers fall into the “Affordable Eco-Friendly” category. These consumers prioritize environmental considerations in their food delivery choices, but they also place significant emphasis on price affordability. This highlights a growing public awareness of environmental issues, while also underscoring the importance of economic viability in consumer decision-making. Therefore, promoting sustainable consumption requires not only raising environmental awareness but also offering cost-effective eco-friendly products to better meet the needs of this mainstream consumer segment.

### 6.2 Implications

According to the above conclusions, this paper offers relevant policy implications. Firstly, based on the results,the Green Takeaway Management Standards could be operationalised through a standardised environmental information system for online food-delivery platforms. The positive preferences and the estimates for lower carbon-footprint options indicate that consumers respond to visible differences in carbon performance. Accordingly, platforms could display carbon-footprint categories at the point of choice using a concise and consistent format, such as the low-, medium-, and high-carbon categories adopted in this study. Because consumers also exhibit positive WTP for degradable and recyclable packaging, the information system should separately disclose packaging materials rather than subsuming them under a general “green” designation. To ensure comparability and credibility, government agencies or authorised third-party institutions should establish common accounting boundaries, classification criteria, and verification procedures for both carbon-footprint and packaging-material claims. A phased pilot could initially be conducted on major food-delivery platforms and in cities with mature digital infrastructure before wider implementation. Such a gradual approach would allow regulators and platforms to assess compliance costs, consumer comprehension, and potential unintended effects before considering broader disclosure requirements. Furthermore, the government should introduce incentives to encourage takeout platforms to integrate the carbon labeling system and provide technical and market support. Platforms that actively promote the integration of the carbon labeling system and encourage merchant participation could be rewarded with tax reductions or subsidies. Additionally, platforms could recommend low-carbon food options through algorithms or offer discounts and rewards for low-carbon options to further motivate consumers to choose more environmentally friendly takeout.

Secondly, environmental information disclosure under the Green Takeaway Management Standards should be designed not merely to increase the visibility of environmental claims, but also to improve consumers’ understanding of specific environmental attributes. The results show that subjective environmental knowledge strengthens preferences for both lower-carbon-footprint food-delivery options and environmentally friendly packaging, whereas pro-environmental attitudes are significantly associated only with preferences for lower-carbon-footprint options. This suggests that general environmental concern may be insufficient when consumers cannot interpret the meaning and implications of specific product attributes. Government agencies should therefore use mass and social media to explain the meaning, practical relevance, and environmental implications of carbon labels and different packaging materials. These efforts should be accompanied by a unified carbon-footprint certification process, under which government-authorised independent third-party institutions evaluate and periodically audit carbon-label and packaging claims to ensure their accuracy, credibility, and comparability. Food-delivery platforms could complement these measures by embedding concise explanations, pop-up prompts, tooltips, or expandable information in the ordering interface without imposing excessive cognitive burdens on users. Building on existing green nudges, such as rewards for declining disposable cutlery, platforms could also use their social features to share low-carbon consumption practices, organise related campaigns, and gradually cultivate consumers’ environmental knowledge and sustainable food-delivery habits. Together, these measures would make the information-disclosure component of the standards more transparent, comprehensible, credible, and enforceable.

Thirdly, the government and food-delivery platforms should jointly adopt differentiated incentives, implementing a series of strategies for different consumer groups to segment the market and promote low-carbon consumption behavior. The latent class results indicate that consumer groups differ substantially in their trade-offs among environmental attributes, price, and service quality. Carbon-sensitive consumers could be encouraged through carbon-point rewards and cumulative emissions-reduction records, while the largest segment-consumers who value both affordability and environmental performance-may respond more effectively to modest discounts, green coupons, or other price incentives combined with carbon labels. Quality-oriented consumers may be more receptive when lower-carbon options are accompanied by reliable platform ratings and service-quality assurances. In addition, platforms should also establish consumer feedback mechanisms to improve carbon-label design and lower-carbon offerings. On the merchant side, government agencies, platforms, and environmental organisations could provide training, calculation tools, and technical support for emissions reduction, sustainable packaging, and environmental information disclosure. A transparent performance-evaluation system could then recognise and appropriately reward merchants that adopt verified carbon labels, sustainable packaging, optimised delivery practices, or lower-carbon energy sources. These differentiated measures would support the implementation of the Green Takeaway Management Standards while ensuring that personalised recommendations remain transparent and expand, rather than restrict, consumers’ available choices.

Finally, the standards should be supported by merchant-side measures that reduce the cost of compliance. Small food-delivery merchants may face difficulties in measuring product-level emissions, sourcing certified packaging, and updating digital menu information. Platforms and local authorities could therefore provide standardised calculation tools, approved packaging catalogues, technical guidance, and transitional support. Merchant performance assessments could incorporate verifiable indicators such as packaging reduction, the use of certified recyclable or degradable materials, and the completeness of environmental information disclosure. Search-ranking advantages or reduced platform service fees could be considered for merchants that meet these criteria, provided that such incentives are transparent and subject to independent verification.

### 6.3 Future research directions

The findings and limitations of this study suggest three directions for future research. Firstly, future studies should examine whether the stated preferences identified in the discrete choice experiment translate into actual purchasing behavior. The present WTP estimates are based on hypothetical choices and may therefore differ from valuations observed in real market settings. Platform-level field experiments, transaction data, or longitudinal designs could be used to test consumers’ responses to carbon-footprint labels under actual purchasing conditions and to examine whether these responses persist with repeated exposure.

Secondly, it’s vital to further assess the external validity and stability of the identified preference patterns and consumer segments. The present sample consists of college students in the Sichuan-Chongqing region, and the magnitude of preferences, WTP, and latent class membership may differ across populations and market contexts. Replication among non-student consumers, different age and income groups, consumers in other regions, and populations in other countries would help determine whether similar preference structures and latent segments emerge beyond the current study setting.

Lastly, greater attention should be given to the design and communication of environmental information. This study varied carbon-footprint levels while holding the traffic-light label format constant, and the results further indicate that subjective environmental knowledge is associated with preferences for both carbon-footprint levels and environmentally friendly packaging. Future research could compare alternative carbon-label formats, such as colour-coded categories, numerical footprints, and comparative framings, and test whether targeted information on packaging materials improves consumers’ understanding and strengthens environmentally preferable choices.

## Data Availability

The datasets presented in this article are not readily available because the datasets generated and analyzed during the current study are not publicly available at this stage due to respondent privacy protection requirements, confidentiality commitments made during the survey process, restrictions under the data use agreement, and arrangements for subsequent research under the project. Requests to access the datasets should be directed to JZ, zhangjian@sicau.edu.cn.
