# Supplementary material for: Delivering sustainable food: exploring college students’ willingness to pay for carbon labels in China via a choice experiment
Source: Front Public Health. 2026 Sep 17;14:1910782. doi: 10.3389/fpubh.2026.1910782 (PMC13627394; doi:10.3389/fpubh.2026.1910782)
Supplement: Supplementary file 1 [file Image_1.pdf]

Supplementary Materials

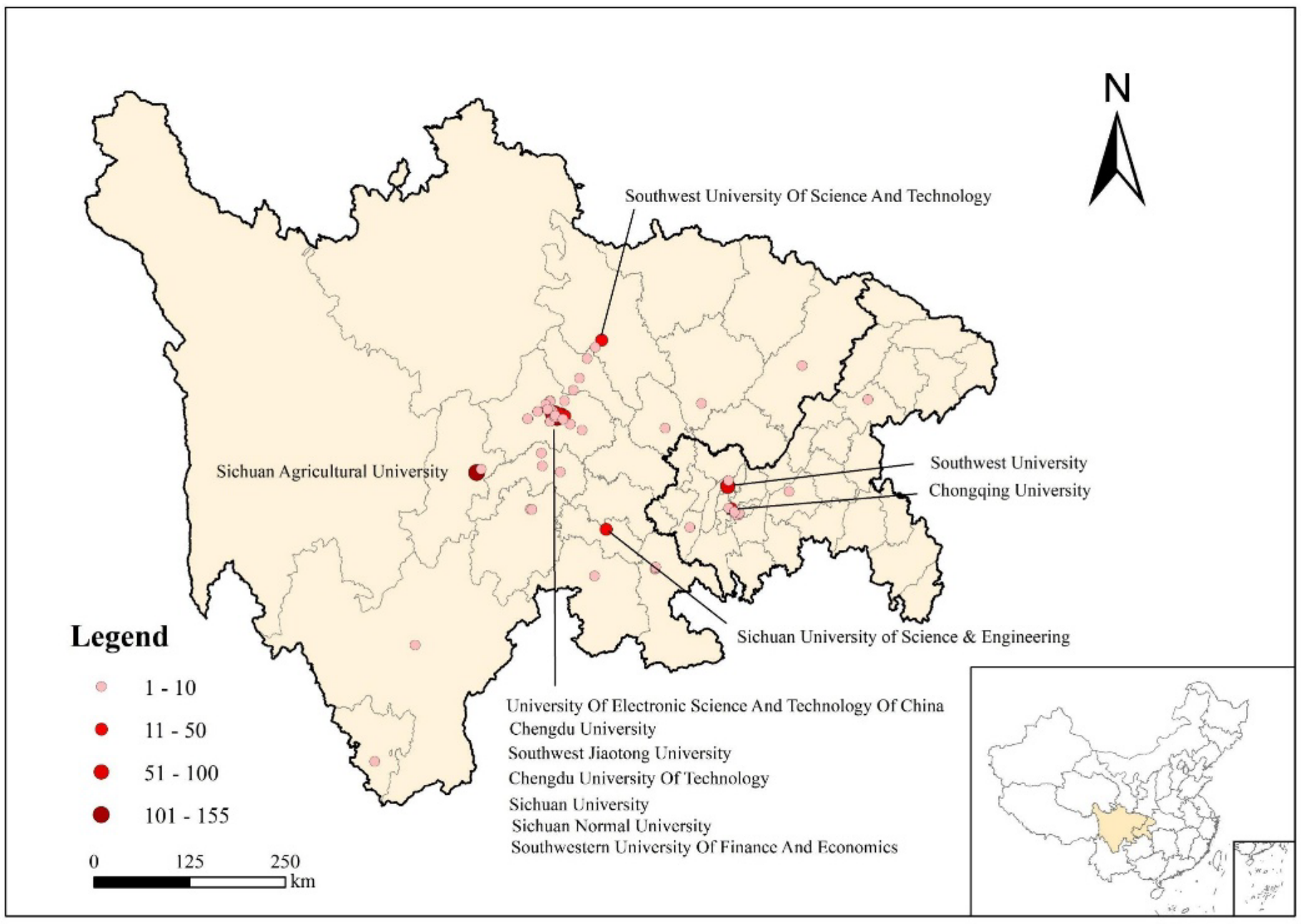

Fig. S1. The distribution of surveyed universities in the Sichuan-Chongqing region.
