# Supplementary material for: Delivering sustainable food: exploring college students’ willingness to pay for carbon labels in China via a choice experiment
Source: Front Public Health. 2026 Sep 17;14:1910782. doi: 10.3389/fpubh.2026.1910782 (PMC13627394; doi:10.3389/fpubh.2026.1910782)
Supplement: Supplementary file 2 [file Table_1.docx]

Table A1 New Ecological Paradigm Scale and Subjective Environment Knowledge Scale

|  | New Ecological Paradigm Scale | Subjective Environment Knowledge Scale |
| --- | --- | --- |
| Question 1 | We are approaching the limit of the number of people the earth can support | I am aware of the classification standards for noise pollution |
| Question 2 | Humans have the right to modify the natural environment to suit their needs | I am knowledgeable about the water consumption of various fabric materials during their production process |
| Question 3 | When humans interfere with nature it often produces disastrous consequences | I understand the comparative energy efficiency of different types of lighting currently available |
| Question 4 | Human ingenuity will insure that we do NOT make the earth unlivable | I am familiar with the criteria for classifying species as endangered |
| Question 5 | Humans are severely abusing the environment | I know the power consumption of various household appliances in standby mode |
| Question 6 | The earth has plenty of natural resources if we just learn how to develop them | I am aware of the energy consumption involved in producing different types of containers, such as plastic and glass bottles |
| Question 7 | Plants and animals have as much right as humans to exist | I can visually determine if soil is affected by salinization based on its appearance |
| Question 8 | The balance of nature is strong enough to cope with the impacts of modern industrial nations | I know the decomposition time of different types of waste |
| Question 9 | Despite our special abilities humans are still subject to the laws of nature | I am informed about the evaluation indicators for the energy-saving capabilities of home appliances |
| Question 10 | The so-called “ecological crisis” facing humankind has been greatly exaggerated | I am familiar with the standards for assessing water quality |
| Question 11 | The earth is like a spaceship with very limited room and resources | I am aware of the potential impacts of acid rain on human life |
| Question 12 | Humans were meant to rule over the rest of nature | I understand the potential health hazards of air pollution to humans |
| Question 13 | The balance of nature is very delicate and easily upset | I am informed about the array of problems that global warming may cause |
| Question 14 | Humans will eventually learn enough about how nature works to be able to control it | I know which modes of daily transport are considered low carbon |
| Question 15 | If things continue on their present course, we will soon experience a major ecological catastrophe | I am aware of the potential health damage caused by noise pollution to humans |
